# miRNA Pattern in Hypoxic Microenvironment of Kidney Cancer—Role of PTEN

**DOI:** 10.3390/biom12050686

**Published:** 2022-05-11

**Authors:** Aleksandra Majewska, Klaudia Brodaczewska, Aleksandra Filipiak-Duliban, Arkadiusz Kajdasz, Claudine Kieda

**Affiliations:** 1 Laboratory of Molecular Oncology and Innovative Therapies, Military Institute of Medicine, 128 Szaserow Street, 04-141 Warsaw, Poland; kbrodaczewska@wim.mil.pl (K.B.); afilipiak1@wim.mil.pl (A.F.-D.); ckieda@wim.mil.pl (C.K.); 2 Postgraduate School of Molecular Medicine (SMM), Warsaw Medical University, 61 Zwirki and Wigury Street, 02-091 Warsaw, Poland; 3 Department of RNA Metabolism, Institute of Bioorganic Chemistry, Polish Academy of Sciences, Noskowskiego 12/14, 61-704 Poznan, Poland; akajdasz@ibch.poznan.pl; 4 Laboratory of Human Molecular Genetics, Faculty of Biology, Institute of Molecular Biology and Biotechnology, Adam Mickiewicz University Poznan, 61-614 Poznan, Poland; 5 Centre for Molecular Biophysics, UPR 4301 French National Centre for Scientific Research CNRS, 45071 Orleans, France

**Keywords:** microRNA, RCC, tumor hypoxia, PTEN

## Abstract

MicroRNAs are post-transcriptional regulators of gene expression, and disturbances of their expression are the basis of many pathological states, including cancers. The miRNA pattern in the context of tumor microenvironment explains mechanisms related to cancer progression and provides a potential target of modern therapies. Here we show the miRNA pattern in renal cancer focusing on hypoxia as a characteristic feature of the tumor microenvironment and dysregulation of PTEN, being a major tumor suppressor. Methods comprised the CRSPR/Cas9 mediated PTEN knockout in the Renca kidney cancer cell line and global miRNA expression analysis in both in vivo and in vitro (in normoxic and hypoxic conditions). The results were validated on human cancer models with distinct PTEN status. The increase in miR-210-3p in hypoxia was universal; however, the hypoxia-induced decrease in PTEN was associated with an increase in miR-221-3p, the loss of PTEN affected the response to hypoxia differently by decreasing miR-10b-5p and increasing miR-206-3p. In turn, the complete loss of PTEN induces miR-155-5p, miR-100-5p. Upregulation of miR-342-3p in knockout PTEN occurred in the context of the whole tumor microenvironment. Thus, effective identification of miRNA patterns in cancers must consider the specificity of the tumor microenvironment together with the mutations of key suppressors.

## 1. Introduction

MicroRNAs (miRNAs, miRs) are small (20–24 nucleotides), non-coding, single-stranded RNA molecules that regulate gene expression at the post-transcriptional level. Their main action consists of suppressing gene expression by recognizing the complementary 3′ untranslated region (UTR) of the target mRNA, which allows its cleavage with subsequent degradation or translation inhibition [1]. It is estimated that about 30% of human genes are regulated by miRNAs [2], making them significant in many basic biological processes including development, cell differentiation, proliferation and apoptosis. Dysregulation of miRNA expression plays a role in the pathogenesis of many diseases including tumors; the impact of altered miRNA expression has been well documented in many types of cancer. Among others, it has been shown to promote aggressiveness [3], metastases [4], vascularization [5] or resistance to treatment [6]. miRNAs have also been proposed as being useful in diagnostics as biomarkers for cancers [7]. Altogether, miRNAs are important potential therapeutic targets in the treatment of cancer, contributing to the need to expand knowledge in this area.

Hypoxia, low, non-physiological oxygen tension is a characteristic feature of the microenvironment of solid tumors. It is well established that low pO_2_ causes pathological vascularization, promotes aggressive phenotypes and increases metastasis [8]. The cellular response to hypoxia leads to changes mainly regulated by hypoxia-inducible factors (HIFs) [9]. Apart from low oxygen tension, the HIF pathway is modulated in a hypoxia-independent manner—HIF-1α (Hypoxia inducible factor 1 alpha), stabilization may result from VHL mutations. Renal cell carcinoma (RCC) are characterized by frequent VHL (Von Hippel-Lindau) mutations—which, by activating the pathways related to HIF-1α and further VEGF (Vascular Endothelial Growth Factor) [10], makes them highly angiogenic tumors [11]. Despite advances in the treatment of kidney cancer using anti-angiogenesis molecular-based therapies as anti-VEGF antibodies or tyrosine kinase inhibitors (TKI) [12], treatment resistance or relapse are a current problem [13]. The development of new, effective therapies and diagnostic markers may focus on miRNAs deregulated in hypoxic RCC, but new research is required to better understand their role in cancer progression and make their regulation a tool to counteract the pathologic microenvironment.

Many of the mechanisms involved in cancer response to hypoxia are related to changes in miRNA expression induced by low oxygen tension [14]. Hypoxia may regulate miRNA biogenesis at the transcriptional level, epigenetic modification, or the activity of enzymes associated with miRNA maturation [15]. It has been proven that hypoxia affects the reduction of Drosh and Dicer levels in cancer cells through the ETS1/ELK1 transcription factors, which leads to dysregulation of miRNA biogenesis and increased tumor progression [16]. A key regulator of hypoxic response—HIF-1α regulates expression of proangiogenic factors, together with miRNAs expression including miR-210, miR-382, miR-224 and others, as reviewed by Peng et al. [17].

In a meta-analysis of a potential miRNA signature panel comparing RCC to normal renal cells, miR-21 and miR-210 were identified as significantly upregulated and miR-141, miR-200c and miR-429 as downregulated [18]. MiR-210, due to its regulation by HIF-1α a, is of particular importance in kidney cancer for the disruption the HIF-1α/VHL complex formation impaired by frequent VHL mutations in RCC. It had been shown that VHL silencing, causing pseudo-hypoxia, also caused higher expression of miR-210 than hypoxia in human RCC cell lines and was elevated in samples from patients with VHL mutation compared to non-mutated tumors [19]. As VHL-dependent regulation of miRNAs is well known in RCC [20], it is important to find the means to overcome such a mutation-related deleterious imbalance by taking advantage of the potential regulation by activation of other tumor suppressors able to control the tumor growth. To this aim we focused on the PTEN (phosphatase and tensin homolog deleted from chromosome 10) tumor suppressor and its miRNA pattern dependence.

PTEN, a tumor suppressor gene, is frequently mutated in many types of tumors [21]. The loss of PTEN in RCC patients is well documented [22]; however, its prognostic value is still debatable [23]. It is a dual specificity phosphatase that dephosphorylates phosphatidylinositol 3,4,5-trisphosphate (PIP3) to phosphatidylinositol 4,5-diphosphate (PIP2), reversing Phosphoinositide 3-kinase (PI3K) action and resulting in the inhibition of the AKT/mTOR pathway, which controls basic cellular processes such as cell growth, metabolism and apoptosis [24]. The activity of PTEN may be of particular importance in hypoxia, as the role of PTEN in the stabilization of HIF-1α has been demonstrated [25].

PTEN expression may be regulated by miRNA, by directly targeting PTEN mRNA (among others: miR-21, miR-221/22, miR-301a), or inducing the hypomethylation of the PTEN promoter (miR-29, miR-101, miR-185) [26]. However, the loss of PTEN may also have other, miRNA-independent backgrounds such as germline and somatic PTEN mutations, genomic deletion or protein-protein interactions [27], but there is a lack of research showing how the loss of PTEN modulates miRNA expression in RCC. In a mouse model of prostate cancer, PTEN loss resulted in a significant increased expression of sixteen miRNAs including miR-155 and miR-132, and a decreased expression of five miRNAs expression (among others: miR-133a and miR-181) [28]. In *Pten*-deficient T-ALL (T-cell acute lymphoblastic leukemia), miR-26b was identified as deregulated in mice [29]. PTEN’s implication in regulation of the tumor growth is strategic for its upstream position controlling the PI3K/AKT/mTOR pathway activation cascade on the one hand, and for its ability to also control the p53 activity on the other hand, making it the decisive supervisor of the biochemical balance in tumor cell development [30]. The status of PTEN is thus a decisive factor in diseases and is becoming highly significant in hypoxia-dependent pathologies. Indeed, it was demonstrated that PTEN is downregulated by hypoxia, the microenvironment hallmark of solid tumors [31], or cardiac pathologies [32,33]. Along the establishment of hypoxic conditions the produced miRNAs are crucial molecular regulators whose composition and action reflect the effect of such selection pressure. Thus, the evaluation cannot be interpreted independently of the hypoxic microenvironmental context that modulates the expression of the cell regulators as factors as well as miRNAs. Here we aimed to determine the miRNA pattern characteristic for RCC in relation to PTEN status, especially taking into account the tumor hypoxic microenvironment.

## 2. Materials and Methods

### 2.1. Cell Lines

Kidney cancer cell lines with different PTEN status were purchased from ATCC (Manassas, VA, USA). PTEN wild-type: murine Renca (Cat# CRL-2947, ATCC, USA), human Caki-1 (Cat# HTB-48, ATCC, Manassas, VA, USA) and PTEN mutant 786-O cell line (Cat# CRL-1932, ATCC, Manassas, VA, USA). All cancer cells were cultured in RPMI-1640 GlutaMax™ medium (Thermo Fisher Scientific, Waltham, MA, USA), with 10% Fetal Bovine Serum (FBS) (Thermo Fisher Scientific, Waltham, MA, USA). Murine brain derived mature endothelial cells (ECs) (MBr MEC FVB) [34], were cultured in OPTI MEM medium (Thermo Fisher Scientific, USA), with 2% FBS (Thermo Fisher Scientific, Waltham, MA, USA). All cell lines and were passaged at 80% confluence by detaching with Accutase solution (Biolegend, San Diego, CA, USA). Cells used in the experiments were Mycoplasma free as assayed with PCR Mycoplasma Test (Biomedica, Piaseczno, Poland) and did not exceed the 15th passage.

### 2.2. CRPISPR/Cas9 Mediated PTEN Knockout in Renca Cells

The CRISPR/Cas9 System was used to knock out the *Pten* expression in Renca cells [35]. Using the Banchling platform (https://www.benchling.com/, accessed on 10 June 2019), two guide RNA (gRNA) sequences targeting the sequence of the *Pten* gene on chromosome 6 were designed: gRNA1: *CCAATTCAGGACCCACGCGGCGG*, gRNA2: *GAACTGTCCTCCCGCCG-CGTGG*.

A commercially available plasmid pSpCas9(BB)-2A-Puro(PX459)V2.0 (Gene Script, Piscataway, NJ, USA) was used in the procedure. Briefly, a single gRNA targeting *Pten* was introduced into the plasmid by ligating oligonucleotides into the GAAGAC site of Bpil. Plasmids for a single gRNA sequence were amplified with competent bacteria, isolated using ZymoPURE™ II Plasmid MidiprepKit (Zymo Research, Irvine, CA, USA), and used to transfect Renca cells.

Renca cells were seeded in a 24-well plate (10^4^ cells per well) 24 h prior to transfection, allowing them to stick to the surface of the well. Five hours before transfection, cells were starved with medium without enrichment of fetal bovine serum. A mixture of gRNA1 and gRNA2 containing plasmids in a 1:1 ratio was used for transfection. Transfections were performed with Lipofectamine 2000 Transfection Reagent (Thermo Fisher Scientific, Waltham, MA, USA) according to the manufacturer’s protocol. Selections of plasmid containing cells were started 5 h after transfection and continued for another 48 h with puromycin at a concentration of 5 µg/mL. A single clone was selected, expanded, and then used for biological assays. PTEN knockout was confirmed by no detection of protein on a western blot and the sequencing of exon 7 fragment, where gRNA was targeting (single nucleotide duplication was detected). Cells transfected with a plasmid lacking gRNA were used as a negative control. Detailed sequencing data of the obtained clones were prepared using Mutation Surveyor^®^ software and are presented in Supplementary Figure S1.

### 2.3. Healthy and Tumor Kidney Cancer Tissue

Commercially available RNA isolates of the human healthy kidney and kidney tumor (TaKaRa, San Jose, CA, USA) were used to verify the potential clinical significance of tested miRNAs. Details about patient samples are described in Supplementary Materials. Total RNA was used for reverse transcription reactions as described below.

### 2.4. Cell Culture Methods in Normoxic and Hypoxic Conditions

**Figure 1:**
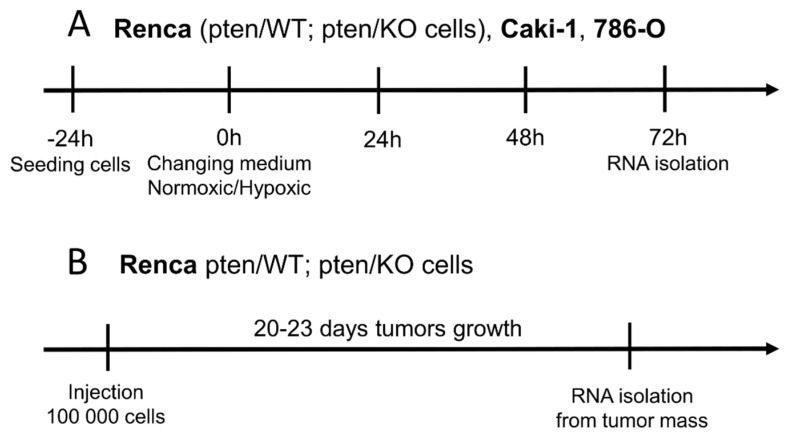
Experimental protocols: (**A**) culture cancer cells in hypoxic and normoxiac conditions (**B**) in vivo experiment obtaining tumors composed of Renca pten/WT or pten/KO cells.

Cancer cells were seeded on standard tissue culture treated flasks (VWR International, Radnor, PA, USA) in the right density (7000 cells/cm^2^ Renca pten/WT pten/KO, 12,000 cells/cm^2^ Caki-1 and 1000 cells/cm^2^ 786-O) and allowed to adhere to the culture surface for 24 h. Media were exchanged with pre-equilibrated normoxic or hypoxic medium and cultured in a standard cell culture incubator (21% pO_2_; 5% CO_2_) or XVivo X3 workstation (Biospherix, Parish, NY, USA) in 1% pO_2_; 5% CO_2_, respectively. After 72 h, cells were harvested using Accutase (Biolegend, San Diego, CA, USA), cell counts and viability were assessed by a Trypan blue exclusion test using a Burker chamber. Collected cells were used for further analysis—protein and RNA isolation. The scheme of the in vitro experiments is presented in Figure 1A.

### 2.5. In Vivo Renca pten/WT and pten/KO Tumor Implantation and Development

BALB/c, six-to-eight-week-old female mice were obtained from the Medical University of Bialystok. Animal care and experimental procedures were approved by the Second Warsaw Local Ethics Committee for Animal Experimentation (no. WAW2/76/2017) and performed following Directive 2010/63/EU regulations. Mice were housed in a controlled environment with a 12 h light/12 h dark cycle with ad libitum access to tap water and full-fledged diet.

Renca cells were implanted in BALB/c mice leg as subcutaneous tumors by injection of a plug constituted by 10^5^ cells in 100 μL Matrigel™ (Corning, NY, USA) diluted in 1:3 in PBS. After 22 days of tumor growth, the mice were sacrificed and fragments of tumor tissue were used to isolate RNA (Figure 1B). The groups consisted of 4 mice and the experiment was performed using two separate sets of animals.

### 2.6. Total RNA Isolation

Total RNA was extracted from 4 × 10^6^ cancer cells cultured in normoxia and hypoxia or 40 mg of tumor tissues using an RNeasy Mini Kit (Qiagen, Hilden, Germany). The samples were freed from DNA using TURBO DNA-free kit (Thermo Fisher Scientific, Waltham, MA, USA) and RNA quality and concentrations were evaluated using the fluorometer Qubit (Qubit RNA BR Assay Kit, RNA with Qubit RNA IQ Assay Kit, Thermo Fisher Scientific, Waltham, MA, USA) according to the manufacturer’s instructions.

### 2.7. Next Generation Sequencing

Total RNA from Renca cells and tumors (pten/WT, pten/KO) was used to prepare libraries and perform high-throughput sequencing by an external service (Lexogen GmbH, Vienna, Austria) with a NextSeq 500 system (Illumina, San Diego, CA, USA). Briefly, RNA concentration was measured by UV-Vis spectrophotometry (Nanodrop 2000c, Thermo Fisher), and RNA integrity was assessed on a Fragment Analyzer System using the DNF-471 RNA Kit (15 nt) (Agilent, Santa Clara, CA, USA). Sequencing-ready libraries were produced using a Small RNA-SeqLibrary Prep Kit by Lexogen (052UG128V0110) per manufacturer instructions. Indexed library preparation was performed to allow for multiplexed sequencing. For library preparation, 100 ng of each provided RNA sample was used. All libraries were analyzed for adapter dimers, size distribution and concentration on a Fragment Analyzer System using the DNF-474 HS NGS Fragment kit (1–6000 bp) (Agilent, Santa Clara, CA, USA). Libraries were pooled in an equimolar ratio. After pooling, 1.3× magnetic bead purification and agarose gel size selection was performed to target miRNA fraction. The concentration and the size distribution of the final lane mix was analyzed by Qubit dsDNA HS assay (Thermo Fisher Scientific, Waltham, MA, USA) and by a Fragment Analyzer system using the DNF-474 HS NGS Fragment Kit (1–6000 bp) (Agilent, Santa Clara, CA, USA). Dilution of the lane mix (2 nm) was denatured and diluted to loading concentration for sequencing on a NextSeq 500 instrument with a SR75 High Output Kit (Illumina). Differentially expressed miRNA (DEmiRNAs) were classified according to −1 < logFC > 1, adjusted *p*-value—FDR (False Discovery Rate) <0.05. NGS data have been deposited in NCBI’s Gene Expression Omnibus [36] and are accessible through GEO Series accession number GSE197301 (https://www.ncbi.nlm.nih.gov/geo/query/acc.cgi?acc=GSE197301, published on 10 May 2022).

### 2.8. Validation of DEmiRNAs Expression by qRT-PCR

The expression of potential DEmiRNAs were analyzed by qRT-PCR both in Renca cells and tumors (pten/WT, pten/KO), as well as human cells with functional and mutant PTEN (Caki-1 and 786-O). Total RNA (10 ng) was used to obtain cDNA using TaqMan™ Advanced miRNA cDNA Synthesis Kit (Thermo Fisher Scientific, CA, USA, USA) according to the manufacturer’s protocol. Briefly, PolyA tailing, adapter ligation and reverse transcription reaction with universal primers were performed. The product of reverse transcription was amplified, then diluted 1:10 and used for the qRT-PCR reaction with TaqMan™ Fast Advanced Master Mix and TaqMan™ Advanced miRNA Assays (listed in Table 1, Thermo Fisher Scientific, Waltham, MA, USA). Reactions were run on Bio-Rad CFX384 qPCR System (BioRad, Hercules, CA, USA), according to the protocol described in Table 2. The relative miRNAs levels were calculated with 2(-Delta C(T)) method, with normalization to the expression of reference miRNAs: miR-16-5p and miR-25-3p. For tissue samples from healthy and kidney cancer, only miR-25-3p was used as reference miRNA.

### 2.9. Prediction of Potential Targets for DEmiRNAs

The target genes for validated DEmiRNAs were assessed using an miRNet (http://www.mirnet.ca/, accessed on 1 December 2021), bioinformatics tool for target prediction based on 11 different databases. The same software was used to assess potential pathways regulated by target genes of DEmiRNAs based on the Function Explorer and KEGG database.

### 2.10. Evaluation of the Expression of Target Genes for DEmiRNAs by qRT-PCR

For cDNA synthesis, 2 µg of total RNA was used for reverse transcription reaction (High-Capacity cDNA Reverse Transcription Kit; Thermo Fisher Scientific, Waltham, MA, USA) and the product was diluted 3×. Real-time PCR was performed using TaqMan™ Gene Expression Master Mix with TaqMan probes or using PowerUp™ SYBR™ Green Master Mix (all from Thermo Fisher Scientific, Waltham, MA, USA, listed in Table 3 and Table 4). Reactions were run on Bio-Rad CFX384 qPCR System or CFX Connect qPCR System (BioRad, Hercules, CA, USA), according to the protocol described in Table 5 and Table 6. The relative mRNA level was calculated with the 2(-Delta C(T)) method, with normalization to the expression of *Actinβ* as a house-keeping gene for mouse models and *GusB* for human models.

### 2.11. Western Blot

Samples were lysed with an RIPA buffer containing Cocktail inhibitors (both Thermo Fisher Scientific, Waltham, MA, USA). 12 µg of total protein, assessed by BCA assay, were solubilized in a Laemmli sample buffer (AlfaAesar, Haverhill, MA, USA), separated on 12% polyacrylamide gel and transferred onto nitrocellulose membranes (BioRad, Hercules, CA, USA). PonceauS staining was performed to detect proteins on the membrane. Non-specific binding was reduced by a blocking step for 2 h in 5% skimmed milk at room temperature. Membranes were incubated overnight at 4 °C in the presence of a solution containing primary antibodies: anti-PTEN (#9549, dilution 1:700, Cell Signaling Technology, Danvers, MA, USA), anti-Vinculin (#SC-59803, dilution 1:1000, Santa Cruz Biotechnology, Dallas, TX, USA), anti-GAPDH (#SC-32233, dilution 1:1000, Santa Cruz Biotechnology, Dallas, TX, USA), anti-pAKT (#MAB887, dilution 1:1000, R&D System, Minneapolis, MN, USA), and then incubated for 2 h in the relevant secondary antibody (Goat Anti-Rabbit IgG Antibody or Horse Anti-Mouse IgG Antibody conjugated with horseradish peroxidase (HRP) (#Pl-1000, #Pl-2000, both used at 1:10,000, from Vector Laboratories, Burlingame, CA, USA). Reactive bands were revealed by reaction with enhanced chemiluminescence substrate (Santa Cruz Biotechnology, Dallas, TX, USA) and visualized using X-ray films (Carestream Health, Rochester, NY, USA). Quantification of the integrated optical density (IOD) of the bands was performed using ImageJ analysis software. In quantitative analysis, the relative IOD of target proteins were normalized to the IOD of Vinculin.

### 2.12. Exosomes Isolation

Exosome isolation from Renca pten/WT cells cultured 72 h in normoxic and hypoxic conditions was performed using ExoQuick-TC (System Bioscience, Palo Alto, CA, USA) according to the manufacturer’s instructions. In brief, conditioned medium was collected and centrifuged at 2000× *g* for 30 min to remove cell residues. ExoQuick-TC exosome precipitation solution was added to the supernatant (in ratio 1:5) and incubated overnight at 4 °C. The suspension was then centrifuged at 1500× *g* for 30 min and a pellet was used to isolate RNA or proteins. The presence of the exosomal TSG101 marker was confirmed on the protein isolate by the western blot method (anti-TSG101 antibody #NBP1-80659, dilution 1:500, Novus Biologicals, Littleton, CO, USA; secondary antibody Goat Anti-Rabbit IgG Antibody, conjugated with horseradish peroxidase (HRP), dilution 1:10,000, Vector Laboratories, Burlingame, CA, USA), the size of the isolated nanoparticles was estimated using the Zeta Viewer Nanoparticle Analyzer (Supplementary Figure S2).

### 2.13. Culture MBr MEC FVB with Conditioned Medium from Renca Cells

MBr MEC FVB immortalized brain endothelial cells as described were seeded on tissue culture flasks (Falcon-Corning, NY, USA) at 3000 cells/cm^2^ and allowed to adhere to the culture surface for 24 h (protocol of the experiment is represented as scheme on Figure 1B). Media were exchanged with pre-equilibrated normoxic or hypoxic medium or mixed (1:1) with conditioned medium from Renca cells from appropriate aerobic conditions and cultured in normoxia and hypoxia for the following 48 h and used for protein isolation.

### 2.14. Statistical Analysis

Each in vitro experiment was performed at least three times in independent biological replicates. The results are shown as a mean ± SEM, where appropriate results are presented as fold change as compared to normoxia. All statistical analyses were performed using GraphPad Prism 9version 9.1.2 for Windows (GraphPad Software, San Diego, CA, USA, www.graphpad.com, accessed on 26 February 2022).

## 3. Results

### 3.1. PTEN Status in Tested Models

**Figure 2:**
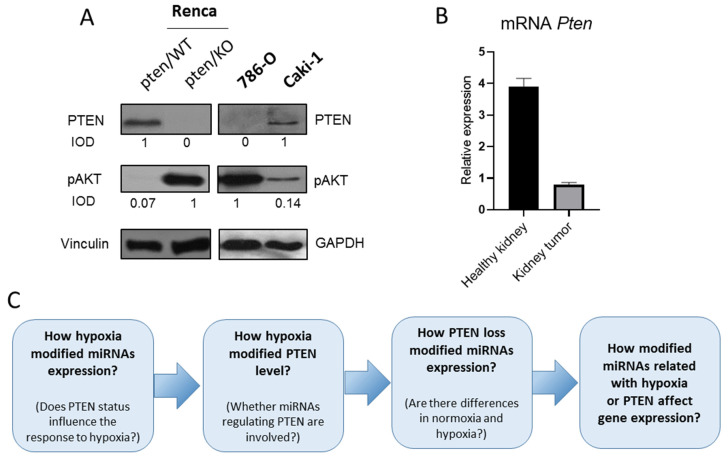
PTEN status in tested models (**A**) PTEN and pAKT levels in kidney cancer cell lines, IOD calculated relative to Vinculin (**B**) Relative to *GusB* expression of *Pten* in RNA isolates from kidney cancer and healthy kidney tissues (**C**) graphical workflow.

To determine the miRNA patterns characteristic for RCC in relation to hypoxia and PTEN status, both in vitro (in normoxic and hypoxic conditions) and in vivo samples from murine kidney cancer model Renca (pten/WT, pten/KO) were analyzed using next generation sequencing. Then, to expand and validate the obtained results by comparison with human species, renal cancer cell lines with functional and mutated PTEN (Caki1 and 786-O respectively) were examined in normoxic and hypoxic conditions. The level of PTEN in all tested cell lines was confirmed by western blot (Figure 2A). Dysregulation of the PTEN activity in 786-O and Renca pten/KO cells is demonstrated by a high level of pAKT (Figure 2A).In a murine PTEN knockout model, p-AKT expression was approximately 14-fold higher than in pten/WT cells, respectively in the human models, the increase was 7-fold. Potential clinical significance of tested miRNAs was marked using commercially available RNA isolates from healthy kidney and kidney tumor, characterized by reduced PTEN levels in cancer tissue as compared to a healthy one (Figure 2B). In all models tested, the expression of miRNA and target genes was assessed in hypoxic conditions and depending on PTEN status in the order described in the graphical workflow (Figure 2C).

### 3.2. Hypoxia-Induced miRNA Signature in Murine Kidney Cancer Models with Different PTEN Status

**Figure 3:**
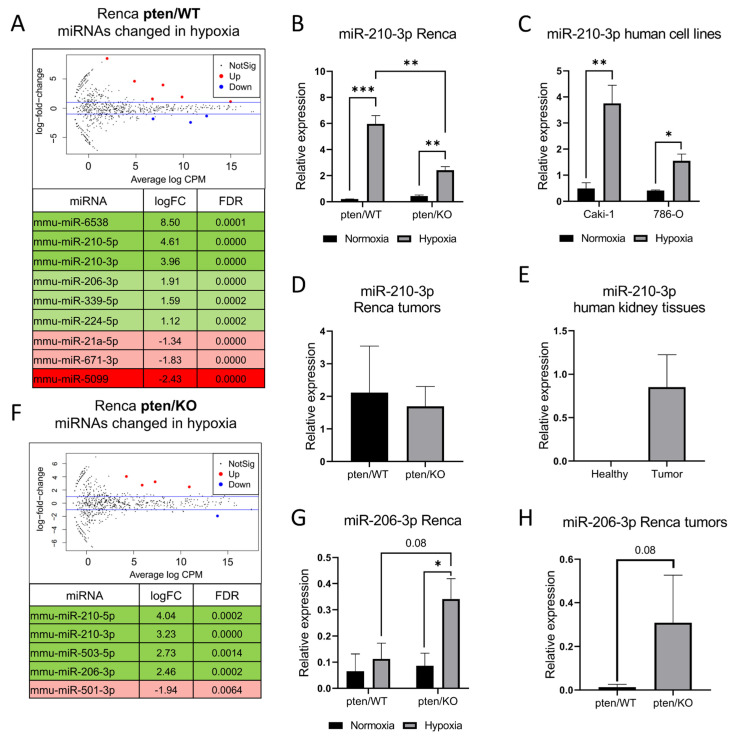
Hypoxia-induced miRNA signature in murine kidney cancer models with different PTEN status (**A**) Volcano plots and list of significantly differentially expressed miRNAs after exposure to hypoxia in Renca pten/WT cells; shades of green represent miRNA upregulated in hypoxia; shades of red downregulated in hypoxia (**B**) Relative to miR-25-3p and miR-16-5p expression of miR-210-3p in cells cultured in normoxic and hypoxic condition: Renca pten/WT and pten/KO (**C**), Caki-1 and 786-O (**D**) Relative to miR-25-3p and miR-16-5p expression of miR-210-3p in tumors derived from Renca pten/WT and pten/KO cells (**E**) Relative to miR-25-3p expression of miR-210-3p in human tissues from healthy kidney and kidney cancer (**F**) Volcano plots and list of significantly differentially expressed miRNA after exposure to hypoxia in Renca pten/KO cells (**G**) Relative to miR-25-3p and miR-16-5p expression of miR-206-3p: in Renca pten/WT and pten/KO cultured in normoxia and hypoxia (**H**) in the tissues from Renca pten/WT and pten/KO tumors, Student’s *t*-test *** *p*-value < 0.001; ** *p*-value < 0.01; * *p*-value < 0.05.

A global miRNA analysis showed only a few changed miRNAs in response to low oxygen tension in both PTEN variants, as presented by volcano plots (Figure 3A,F). In hypoxic Renca pten/WT, nine deregulated miRNAs reached statistical significance (six up-regulated, three down-regulated) compared to normoxic cells (Figure 3A). However, even fewer changes were noted in pten/KO cells in response to low pO_2_—5 DEmiRNAs of which only one was downregulated (Figure 3F). To verify the standard hypoxic response, miR-210-3p and miR-206-3p expression, which was up-regulated in both PTEN variants, was further studied using additional models. qRT-PCR analysis confirmed that miR-210-3p is upregulated in hypoxia regardless of the status of PTEN, however in PTEN mutants (Renca pten/KO and 786-O) this increase was considerably lower than in PTEN wild-type cells (Renca pten/WT, Caki-1) (Figure 3B,C). No difference was noticed between miR-210-3p levels in the tissues from pten/WT and pten/KO tumors, which suggests that the regulation of miR-210-3p occurs in a PTEN independent manner (Figure 3D). As expected, miR-210-3p expression was at an undetectable level in tissue isolated from a healthy kidney and was strongly expressed in kidney tumor tissue (Figure 3E). In turn, miR-206-3p expression measured by qPCR was upregulated by hypoxia only in pten/KO cells, not in pten/WT (Figure 3G). These results are consistent with NGS analysis; the changes in miR-206-3p expression in Renca pten/WT was characterized by lower logFC (1.9) (Figure 3A), while in Renca pten/KO logFC = 2.46 (Figure 3F). Increasing miR-206-3p after exposure to low pO_2_ in pten/KO cells resulted in a strong tendency to change the expression of this miRNA between pten/WT and pten/KO cells in hypoxia (*p*-value = 0.08) (Figure 3G). This tendency was also observed in in vivo samples, which reflect the hypoxic conditions (Figure 3H).

### 3.3. Identification of PTEN-Related miRNA in Hypoxic Conditions

**Figure 4:**
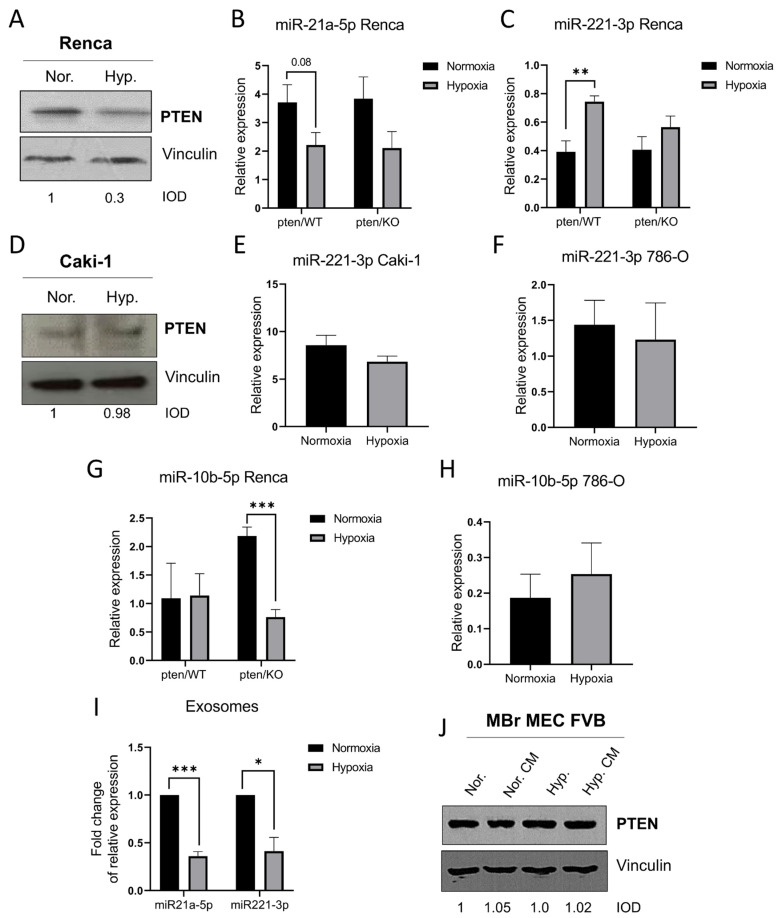
PTEN-related miRNAs in hypoxic conditions (**A**) PTEN detection by western blot in Renca pten/WT cells cultured in normoxic and hypoxic condition, IOD calculated relative to Vinculin (**B**) Relative to miR-25-3p and miR-16-5p expression of miR-21a-5p and (**C**) miR-221-3p in Renca pten/WT and pten/KO cells cultured in normoxia and hypoxia (**D**) PTEN detection by western blot in Caki-1 cells cultured in normoxic and hypoxic condition, IOD calculated relative to Vinculin (**E**) Relative to miR-25-3p and miR-16-5p expression of miR-221-3p in Caki-1 cells, and (**F**) in 786-O cells cultured in normoxia and hypoxia (**G**) Relative to miR-25-3p and miR-16-5p expression of miR-10b-5p in Renca pten/WT and pten/KO cells and (**H**) 786-O cells cultured in normoxia and hypoxia (**I**) Fold change of relative to miR-25-3p and miR-16-5p expression of miR-21a-5p and miR-221-3p in exosomes isolated from Renca pten/WT cells cultured in normoxia and hypoxia (**J**) PTEN detection by western blot in MBr MEC FVB cells cultured in normoxic and hypoxic condition with or without conditioned medium (CM) from Renca pten/WT cells, IOD calculated relative to Vinculin, Student’s *t* test *** *p*-value < 0.001; ** *p*-value < 0.01; * *p*-value < 0.05.

NGS analysis showed miR-21a-5b to be downregulated in response to hypoxia in pten/WT cells. As miR-21a is a well-known regulator of PTEN, and its expression was further validated in the context of the hypoxia-induced decreased PTEN level observed in Renca pten/WT cells (Figure 4A). We confirmed the strong tendency to downregulate miR-21a-5p in wild-type cells in response to hypoxia, but the same tendency was observed in pten/KO (Figure 4B). This may suggest that downregulation of miR-21a-5b is not involved in the modulation of PTEN levels in our model (the same pattern in pten/WT and pten/KO cells). Moreover, miR-21a is known to directly degrade *Pten* mRNA, so its decrease does not correlate with a decreased PTEN level.

Thus, we checked whether other miRNAs affecting PTEN described in the literature are changed by hypoxia. miR-221-3p was upregulated in low pO_2_ only in pten/WT cells, while in the absence of PTEN, the level was stable between oxygen conditions (Figure 4C). A different response to hypoxia in pten/WT and pten/KO cells in the context of miR-221-3p expression may suggest its effect on hypoxia-dependent PTEN downregulation in pten/WT cells. Since such changes were not observed in the human PTEN wild-type kidney cancer model Caki-1, no increase in miR-221-3p (Figure 4E) in hypoxia resulted in a stable level of PTEN between oxygen conditions (Figure 4D). In a human PTEN mutant model, 786-O miR-221-3p was also stable between oxygen condition (Figure 4F).

Another tested PTEN-related miRNA was miR-10b-5p, which was stable between the oxygen condition in Renca pten/WT cells, but was significantly downregulated in hypoxia in pten/KO cells (Figure 4G)., indicating that miR-10b-5p may be involved in the altered hypoxia response due to the complete loss of PTEN. However, this trend has not been confirmed in a human RCC model with non-functional PTEN (Figure 4H).

We also checked the expression of PTEN-related miRNAs in exosomes secreted by Renca pten/WT cells to determine the impact of these changes on other components of the tumor microenvironment (TME). Expression of miR-21a-5p was lower in hypoxic exosomes compared to normoxic ones, which corresponds to the level of this miRNA in cells (Figure 4I). At the same time, upregulation of miR-221-3p was not transferred to exosomes secreted by Renca cells in hypoxic conditions. (Figure 4I). Additionally, exosomes secreted by Renca cells, present in the conditioned medium, did not affect PTEN levels in brain-derived healthy endothelial cells (MBr MEC FVB) (Figure 4J). miR-10b-5p was below detection in samples from exosomes (data not shown).

### 3.4. PTEN-Dependent miRNA Pattern

**Figure 5:**
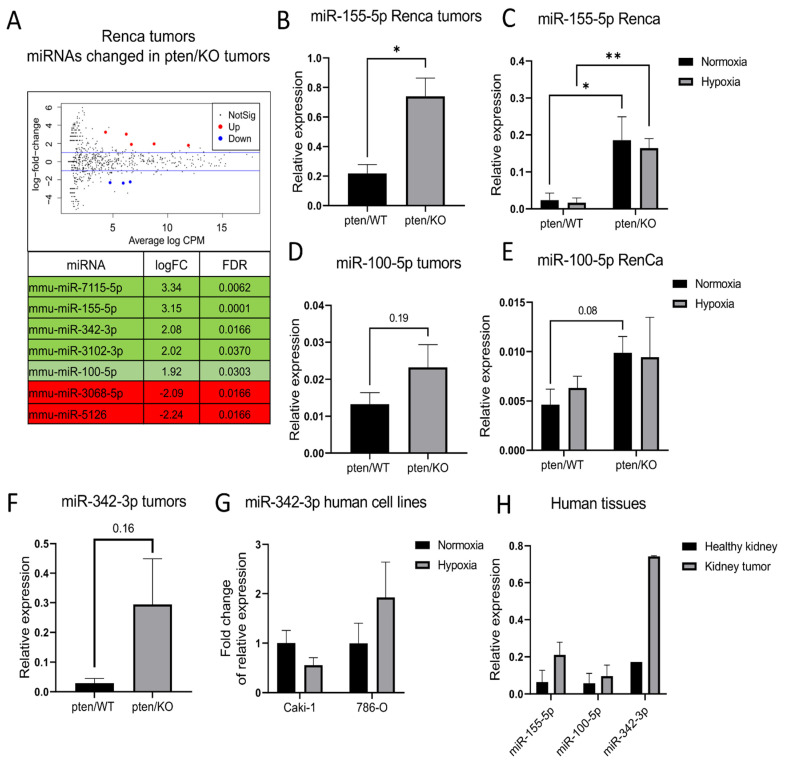
PTEN-depended miRNA pattern in RCC models (**A**) Volcano plots and list of significantly differentially expressed miRNAs after in pten/KO tumors; shades of green represent miRNA upregulated in pten/KO cells; shades of red downregulated in pten/KO cells (**B**) Relative to miR-25-3p and miR-16-5p expression of miR-155-5p in tumors derived from Renca pten/WT and pten/KO cells (**C**) Relative to miR-25-3p and miR-16-5p expression of miR-155-5p in Renca pten/WT and pten/KO cells cultured in normoxic and hypoxic condition (**D**) Relative to miR-25-3p and miR-16-5p expression of miR-100-5p in tumors derived from Renca pten/WT and pten/KO cells (**E**) Relative to miR-25-3p and miR-16-5p expression of miR-100-5p in Renca pten/WT and pten/KO cells cultured in normoxic and hypoxic condition (**F**) Relative to miR-25-3p and miR-16-5p expression of miR-342-3p in tumors derived from Renca pten/WT and pten/KO cells and in tumors derived from Renca pten/WT and pten/KO cells (**G**) Fold change of relative to miR-25-3p and miR-16-5p expression of miR-342-3p in Caki-1 and 786-O cells cultured in normoxia and hypoxia (**H**) Relative to miR-25-3p expression of miR-155-5p, miR-100-5p and miR-342-3p in human tissues from healthy kidney and kidney tumor, Student’s *t*-test ** *p*-value < 0.01; * *p*-value < 0.05.

We next verified how the total loss of PTEN affects the miRNA pattern in RCC in normoxic and hypoxic conditions, as well as in in vivo samples. Surprisingly, NGS analysis showed no significantly deregulated miRNAs in vitro samples from pten/KO cells compared to the wild-type in both normoxic and hypoxic conditions (Supplementary Figure S3A,B). However, despite the lack of altered expression in vitro, in vivo samples revealed some DEmiRNAs. In tumors derived from KO cells compared to WT 7, deregulated miRNAs were identified (5 up, 2 down) (Figure 5A). In in vitro samples, these miRNAs did not achieve statistical significance (FDR > 0.05), as demonstrated in Supplementary Figure S3A,B. Next we validated the expression of miR-155-5p, miR-100-5p and miR-342-3p, since they were shown to be important in the PTEN mediated pathways, namely miR-155-5p and miR-100-5p, were the most efficiently changed in vivo together with miR-7115 that had no validated target, as yet.

MiR-155-5p was significantly upregulated in pten/KO tumors compared to the wild-type as confirmed by qPCR (Figure 5B). The same was observed in in vitro samples both in hypoxic and normoxic conditions (Figure 5C). Interestingly, the hypoxia-induced decrease in PTEN in Renca cells was insufficient to induce miR-155-5p expression. The increase in miR-100-5p expression observed by NGS was not validated by qRT-PCR from in vivo samples, however in in vitro experiments the tendency was strong (*p*-value = 0.08) (Figure 5D,E). The same as miR-155-5p, expression of miR-100-5p was independent of oxygen conditions. miR-342-3p was not detected in Renca in vitro samples (data not shown), however in Renca pten/KO tumors it tended to be upregulated compared to pten/WT (Figure 5F). Experiments on human models showed that miR-342-3p regulation occurs in a hypoxia-independent manner regardless of the PTEN status (Figure 5G). In kidney cancer tissue, characterized by the decreased expression of PTEN compared to healthy kidney, miR-155-5p and miR-342-3p were upregulated, and no difference in expression of miR-100-5p was observed (Figure 5H).

### 3.5. Identification of Potential Hypoxia- and PTEN-Dependent miRNAs Targets

**Figure 6:**
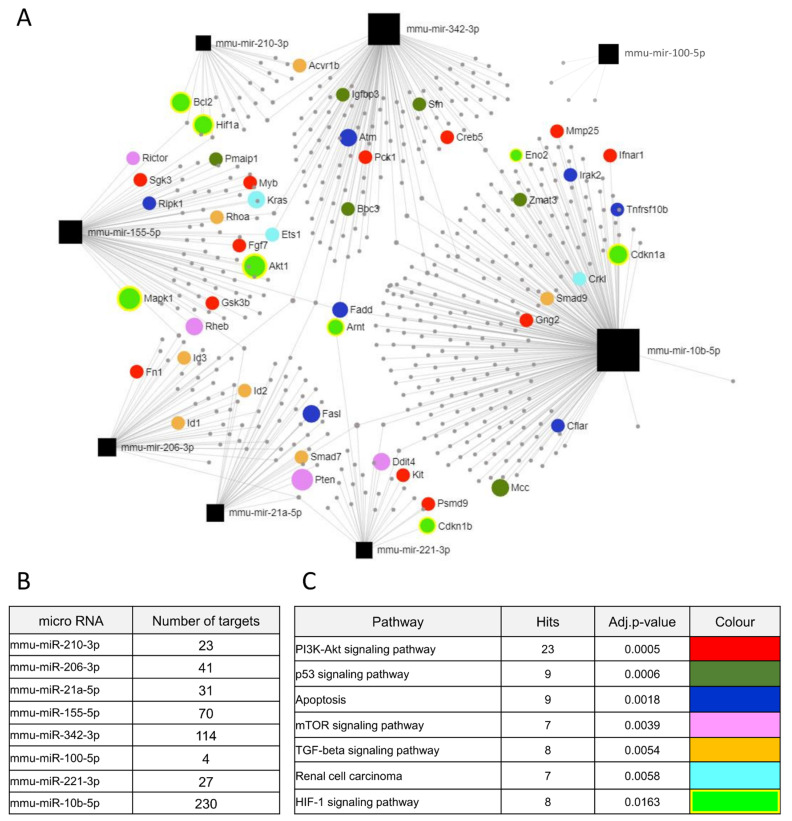
The predicted target genes of DEmiRNAs (**A**) Network of target gene, with marked genes involved in potentially dysregulated signaling pathways, predicted using the miRNET software (**B**) Number of target genes for each DEmiRNA (**C**) Potentially deregulated signalling pathways for predicted target genes analyzed through susceptibility genes enrichment.

**Figure 7:**
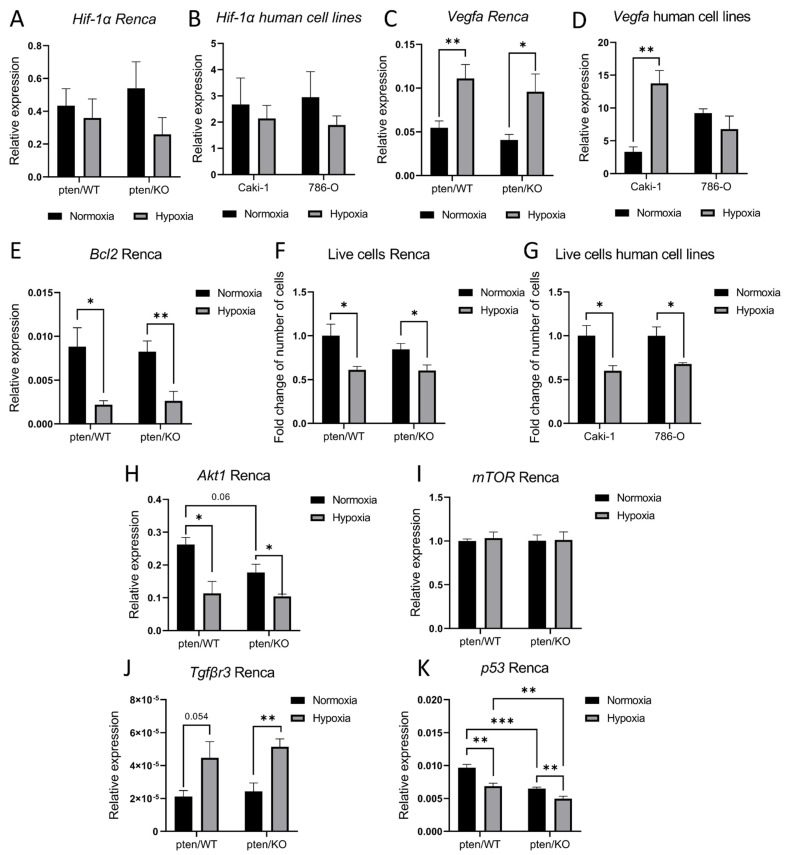
Expression of predicted target genes in RCC models (**A**) Relative to *Actinβ* expression of *Hif-1α* in Renca pten/WT ptenKO cells cultured in normoxia and hypoxia (**B**) Relative to *GusB* expression of *Hif-1α* in Caki-1 and 786-O cells cultured in normoxia and hypoxia (**C**) Relative to *Actinβ* expression of *Vegfa* in Renca pten/WT ptenKO cells cultured in normoxia and hypoxia (**D**) Relative to *GusB* expression of *Vegfa* in Caki-1 and 786-O cells cultured in normoxia and hypoxia (**E**) Relative to *Actinβ* expression of *Bcl2* in Renca pten/WT ptenKO cells cultured in normoxia and hypoxia (**F**) Fold change of number of live cells collected form normoxic and hypoxic culture: Renca pten/WT pten/KO (**G**) and Caki-1, 786-O (**H**) Relative to *Actinβ* expression of *Akt1* (**I**) *mTOR* (**J**) *Tgfβr3* (**K**) *p53* in Renca pten/WT ptenKO cells cultured in normoxia and hypoxia, Student’s *t*-test *** *p*-value < 0.001; ** *p*-value < 0.01; * *p*-value < 0.05.

Next, we identified the potential targets of miRNAs that were validated and significantly changed either by hypoxia or PTEN deregulation. Figure 6A presents networks of potential target genes, showing the number of targets for each miRNA (Figure 6B), as well as the signaling pathways related to the appointed target genes (Figure 6C). They are strongly involved in the PI3K/AKT pathway regulated by PTEN or HIF-1α signaling regulated by hypoxia, but also in apoptosis and the TGF-β signaling pathway. To establish whether deregulation of the tested miRNAs affects the expression of genes, we checked the levels of transcripts of some of the targets. We checked the expression of *Hif-1α* as the main modulator of hypoxic response associated with miR-210-3p activity, but no changes were observed in all tested cell lines (Figure 7A,B). However, *Vegfa*, which is a downstream target of HIF-1α, was upregulated in PTEN wild-type Renca and Caki-1 cell lines in response to hypoxia. In Renca pten/KO this increase was weaker than in Renca pten/WT, and we did not observe an increase in PTEN mutant 786-O cells (Figure 7C,D), which reflects the results of miR-210-3p expression. As the pathway associated with apoptosis was altered in the context of the DEmiRNAs, we checked the expression of antiapoptotic *Bcl2*, which is also associated with HIF-1a signaling, and regulated by miR-210-3p. *Bcl2* was downregulated in hypoxia in both PTEN variants (Figure 7E), and this was correlated with a decreased number of live cells in all tested cell lines (Figure 7F,G). PTEN knockout caused upregulation of miR-155-5p, and we assessed two potential targets of this miRNA: *Akt1* and *mTOR*. *Akt1* tended to be downregulated in Renca pten/KO compared to the wild-type (Figure 7H); however, hypoxia also caused downregulation of *Akt1*, what may suggest that expression is also regulated independently of miR-155-5p, which was stable in pten/WT cells in normoxic and hypoxic condition (Figure 5C). Moreover, no change was observed in *mTOR* expression between both PTEN variants an oxygen level (Figure 7I). Due to the altered expression of miR-21a-5p in response to hypoxia and significant changes in the TGF-β signaling pathway, we assessed the level of *Tgfβr3*, which is modulated by miR-21a-5p. *Tgfβr3* was upregulated in hypoxia in both PTEN variants (Figure 7J). *p53,* which may also be regulated by miR-21a-5p, miR-10b-5p and miR-221-3p, is downregulated by hypoxia and in pten/KO independently (Figure 7K).

## 4. Discussion

PTEN is a tumor suppressor that is frequently mutated in cancers. The frequency of PTEN alterations is around 8% for all cancers, but for some, like endometrial cancer or gliomas, it is almost as high as for p53 mutations [37,38]. Corroborating the limited mRNA hypoxic signatures in cancer cells [39,40] after exposure to hypoxia, we identified only nine differentially expressed miRNA in Renca PTEN wild-type cells as compared to the liver cancer response to hypoxia by 165 altered miRNAs [41]. PTEN knockout altered the expression of seven miRNAs in tumor samples. Similarly, few changes were observed in prostate cancer of a PTEN knockout mouse model [28].

Exposure to hypoxia increased miR-210-3p expression in both PTEN variants. MiR-210 expression increase in low pO_2_ is a well-known and general effect [42]. In RCC patients, miR-210 was one of the five most changed miRNAs in tumors [43], as confirmed here. MiR-210 is induced by HIF-1α, although we observed no difference in *Hif-1α* expression, which might be due to a prolonged exposure to low pO_2_ (72 h), since the downstream *Vegfa* gene expression was increased. This could be partly mediated by miR-210, reported to be proangiogenic [44,45]. However, in 786-O, hypoxia did not upregulate *Vegfa* expression, despite the miR-210-3p increase. As these cells are characterized by VHL mutation, it is clear that a pseudo hypoxic stress is installed and may prevent the miR-210-3p and *Vegfa* sensitivity to hypoxia itself. Apoptosis is also regulated by miR-210-3p. Indeed, fewer viable cells were collected upon hypoxia, coinciding with the downregulation of the Bcl2-antiapoptotic protein, shown to be regulated by this miRNA in neuroblastoma or in the spermatocyte cell line [46,47].

To our knowledge, this is the first study to show the effect of PTEN knockout on miR-210-3p demonstrated by the lower increase of miR-210-3p expression in low pO_2_ in cells with mutant PTEN compared to WT cells. This confirms a role for PTEN in regulating cell responses to low pO_2_ [48]. Together with weaker miR-210-3p upregulation, we observed the induction of miR-206-3p in pten/KO cells in response to hypoxia. VEGF is a target for miR-206, and a negative correlation between miR-206 levels and VEGF expression has been proven in human renal cancer lines [49]. VEGF is differentially regulated by miR-210 (up-regulation) and miR-206 (down-regulation); however, this did not presently result in a disturbed induction of gene expression; in response to hypoxia pten/KO VEGF upregulation was as efficient as in wild-type cells. miR-206 is suggested to be a tumor suppressor [50,51]; it inhibits cancer cell growth and metastasis. MiR-206 was downregulated in RCC compared to non-tumor tissues and in cell lines (Caki-1 and Caki-2) compared to normal human kidney cells [49]. Surprisingly, in PTEN mutated cells, miR-206-3p was elevated in response to hypoxia, suggesting lower aggressiveness. MiR-206 was reported to indirectly regulate the MAPK-PI3K/Akt pathway [50], therefore this miRNA can serve as a regulator of pAKT accumulation upon PTEN mutation. The direct link between miR-206-3p and PTEN could not be established since its activation was a response to hypoxia in pten/KO cells, while PTEN mutation only was not enough to activate miR-206-3p.

At the same time, hypoxia reduced the miR-10b-5p level that was strongly induced in normoxic pten/KO. MiR-10b-5p is an oncomiR, as it promotes cell proliferation, protects against apoptosis [52], and mediates cancer progression [53]. It targets PTEN mRNA [54] leading to the activation of the PI3K/AKT pathway. In our study, cells with dysfunctional PTEN had a higher expression of this miRNA than pten/WT cells, but its level in pten/KO cells was reduced by hypoxia as compared to normoxic controls. Whether a reciprocal regulation of miR-10b by PTEN exists needs to be evaluated, but it might be that in our model miR-10b induction by PTEN-KO could be partially mediated by p53 downregulation [55]. Indeed, PTEN/p53 interaction is described on a protein level [30]; however, they might also share miRNAs regulatory pathways.

Although miR-206-3p and miR-10b-5p were not affected by hypoxia in pten/WT cells, they were differently modulated by low pO_2_ in PTEN mutated cells. This points to the oxygen-dependent role of PTEN as a tumor suppressor, as the observed PTEN inactivation is more frequent in hypoxic tumors [56]. Additionally, the modulation of miRNAs response to hypoxia by PTEN inactivation is of interest for a potential therapeutical approach. miR-206-3p was shown to indirectly repress ABCB1, a multidrug resistance gene [57], and was shown to reduce Cisplatin resistance in gastric cancer [58]. Also, Wang et al. observed that this miRNA increases cancer response to radiotherapy [59]. miR-10b-5b was also shown to alter cancer response to therapy; it mediates resistance to 5-fluorouracil (5-FU) by promoting cancer cell survival [60] and decreases sensitivity to radiotherapy [61]. Therefore, the miRNA profile of hypoxic PTEN mutated cells identified in our study may suggest the promotion of a therapy-sensitive phenotype of tumor cells.

On the other hand, reduced miR-210 levels were observed in cells resistant to 5FU, which promoted their DNA repair and metabolic adaptation to drug treatment [62]. Therefore, the modulated response to hypoxia by PTEN dysregulation, as observed by levels of miR-206-3p, miR-10b-5p and miR-210-3p in this study, can alter the induction of drug resistance pathways. Knowing that reduction [63] or inactivation [64] of PTEN and also hypoxia can increase cancer cell resistance to chemo- and radiotherapy [65], information that PTEN dysregulation disturbs miRNA response to low pO_2_ might serve as a cue for redefining treatment selection in hypoxic PTEN mutated tumors.

MiRNAs regulated by PTEN mutation independently of oxygen level included: miR-155-5p, miR-100-5p, and miR-342-3p. In our research, miR-155-5p was significantly upregulated in pten/KO cells and tumors and was not affected by the oxygen level. In a mouse prostate cancer model with PTEN mutation, the same miRNA was the most upregulated. PTEN is a target for miR-155 [66], so accumulation of miR-155-5p in pten/KO cells may be related with the lack of target. On the other hand, analysis based on miRNet software showed that *Akt1* could also be a target of miR-155-5p. *Akt1* was decreased after PTEN knockout, but hypoxia also caused a decrease in *Akt1* expression independently of PTEN which, together with a stable miR-155-5p level between normoxia and hypoxia in pten/WT and pten/KO cells, may indicate no influence of miR-155-5p on *Akt1* level or other hypoxia-dependent mechanisms regulating *Akt,* not related with miR-155-5p. Other miRNAs that tended to be deregulated in PTEN mutant cells were miR-100-5p and miR-342-3p. miR-100-5p overexpression is strongly associated with tumor progression and adverse clinical outcomes in RCC patients [67]. Although in ovarian cancer the key relationship between miR-100 and the mTOR pathway was confirmed [68], in our RCC model the tendency to increase miR-100-5p after PTEN knockout was not sufficient to change *mTOR* expression. More recently, miR-342-3p was identified as a significant immune-related miRNA, with elevated levels in RCC associated with poorer patient survival [69]. Here, miR-342-3p was not detected in in vitro samples but tended to be upregulated in pten/KO tumors as in kidney tumors. This may indicate the significance of miR-342-3p for immunological components of the tumor microenvironment and these aspects of cancer progression might be deregulated in PTEN mutated tumors.

Other miRNAs modulating PTEN expression in cancer have been identified. We proved that the upregulation in miR-221-3p expression may be involved in PTEN level modulation in low pO_2_, as there was no increase in miR-221-3p in pten/KO hypoxic cells. Modulation of PTEN expression by miR-221 was also described in gastric carcinoma [70], however in the human RCC model Caki-1, no changes were observed in both PTEN level and miR-221-3p expression. This sheds light on the diversity of the miRNA pattern, even in the same tumor types, and points to the need to interpret miRNA expression in a personalized way [71]. Upregulation of miR-221-3p in a mouse kidney cancer model was not transferred through exosomes to cells of TME and we did not observe changes in PTEN expression in mature brain-derived endothelial cells, but in cervical cancer, cell-secreted exosomal miR-221 promoted angiogenesis through Thrombospondin-2 [72]. miR-21a, also targeting PTEN, is described as one of the most upregulated in RCC compared to healthy kidney, which was confirmed by our results performed on total RNA from RCC samples. Surprisingly, miR-21a-5p was downregulated in hypoxia in both PTEN variants in Renca cells, and does not seem to regulate the PTEN levels in a pten/WT model. However, others also showed miR-21a downregulation in response to hypoxia [73], therefore it seems that the miR-21a response to low pO_2_ depends on cancer type [74]. To establish what could be the role of miR-21a-5p inhibition, we looked for targets other than PTEN for this miRNA. Based on miRNet analysis, we demonstrated that *Tgfβr3* may be a target for miR-21a-5p, as confirmed in a glioblastoma model [75]. In response to hypoxia and a simultaneous miR-21a-5p decrease, *Tgfβr3* was upregulated in both PTEN models. The overexpression of *Tgfβr3* in nasopharyngeal carcinoma cells has been demonstrated to increase apoptosis through the downregulation of Bcl2 [76]. This suggests that in some cancers the other effects of miR-21a-5p than its PTEN-regulatory role prevail in response to hypoxia.

## 5. Conclusions

We showed hypoxia-/PTEN-dependent miRNA patterns in RCC and their effects on target gene expression. We confirmed the upregulation of miR-210-3p and miR-221-3p but the downregulation of miR-21a-5p in response to low pO2. Loss of PTEN caused limited miRNA expression modifications, mostly oncomiR-155 upregulation, miR-100-5p and miR-342-3p induction. However, PTEN mutation changed the response to hypoxia. The increase in miR-210-3p is weaker than in cells with functional PTEN; expression of miR-206-3p (suppressor) was upregulated with a simultaneous decrease in miR-10b-5p expression (oncogene), suggesting compensation to the lower aggressiveness of hypoxic pten/KO vs pten/WT cells. PTEN mutation annihilated miR-221-3p responsiveness to hypoxia by induced expression in PTEN-functional cells. Our study shows that miRNAs regulated by/responding to TME (hypoxia) and tumor mutation status (PTEN) might interact and mutually regulate cancer responses. Simultaneously, we demonstrated that low pO_2_ can regulate PTEN levels and miRNA may be involved, showing the complexity of the relationship between the various features of TME. The identification of miRNAs that are modulated by distinct variables might allow for a better understanding of the mechanisms of the pathogenesis of kidney cancers and contribute to the development of effective therapies.

## Figures and Tables

**Table 1:** List of TaqMan probes used to detect miRNA expression.

miRNA	Assay Number
miR-210-3p	mmu481343_mir
miR-206-3p	mmu481645_mir
miR-100-5p	mmu478224_mir
miR-155-5p	mmu480953_mir
miR-100-5p	478224_mir
miR-155-5p	483064_mir
miR-342-3p	mmu481074_mir
miR-221-3p	mmu481005_mir
miR-10b-5p	mmu478494_mir
miR-21a-5p	mmu482709_mir
miR-25-3p	mmu483226_mir
miR-16-5p	mmu482960_mir

**Table 2:** qRT-PCR conditions—TaqMan™ Fast Advanced Master Mix (miRNA detection).

Step	Temperature	Time	Cycle
Enzyme activation	95 °C	20 s	1
Denature	95 °C	1 s	40
Anneal/Extend	60 °C	20 s	

**Table 3:** List of TaqMan probes used to detect gene expression.

Gene	Taq Man Probes
*Hif-1α*	Mm00468869; Hs00153153
*Vegfa*	Mm00437306; Hs00900055
*Akt1*	Mm00437306
*Tp53*	Mm01731287
*mTOR*	Mm01731287
*Pten*	Hs02621230
*Actinβ*	Mm02619580
*GusB*	Hs00939627

**Table 4:** List of primers sequences used to detect gene expression.

Gene	Primers Sequences
*Bcl2*	F: GACTGAGTACCTGAACCGGC
	R: AGTTCCACAAAGGCATCCCAG
*Tgfβr3*	F: AGTGCTCTGAGTGCTCCCTA
	R: TACTCCCACACAGGGGAGAC
*Actinβ*	F: CCTAGGCACCAGGGTGTGA
	R: GTTGGCCTTAGGGTTCAGGG

**Table 5:** qRT-PCR conditions TaqMan™ Gene Expression Master Mix.

Step	Temperature	Duration	Cycle
UNG incubation	50 °C	2 min	Hold
Polymerase Activation	95 °C	10 min	Hold
Denature	95 °C	15 s	40
Anneal/extend	60 °C	15 s	

**Table 6:** qRT-PCR conditions PowerUp™ SYBR™ Green Master Mix.

Step	Temperature	Duration	Cycle
UDG activation	50 °C	2 min	Hold
Dual-Lock™ DNA polymerase	95 °C	2 min	Hold
Denature	95 °C	15 s	40
Anneal	60 °C	45 s	
Extend	60 °C	1 min	

## Data Availability

All data generated or analyzed during this study are included either in this article or in the Supplementary Figures. The data that support the findings of this study are available from the corresponding author upon reasonable request.

## Supplementary Materials

The following supporting information can be downloaded at: https://www.mdpi.com/article/10.3390/biom12050686/s1, Figure S1: Results of sequencing—PTEN knockout clones; Figure S2: Size of nanoparticles isolated with the ExoQuick Buffer from Renca cells conditioned medium (A) normoxic (B) hypoxic, measured using Zeta Viewer Nanoparticle Analyzer (C) level of exosomal marker TSG101 in samples of isolated nanoparticles; Figure S3: Volcano plots of DEmiRNA between Renca pten/WT and pten/KO cell in (A) normoxic (B) hypoxic conditions. Listed miRNAs significantly changed in pten/KO in vivo samples compared to pten/WT.
