# Supplementary material for: miRNA Pattern in Hypoxic Microenvironment of Kidney Cancer—Role of PTEN
Source: Biomolecules. 2022 May 11;12(5):686. doi: 10.3390/biom12050686 (PMC9138332; doi:10.3390/biom12050686)
Supplement: Supplementary file 1 [file biomolecules-12-00686-s001.zip › biomolecules-1636547-supplementary.pdf]

Supplementary Materials

Commercially available RNA isolates were purchased from TaKaRa, San Jose, CA, USA.
2 independent vials of RNA isolates were used in each experiment.
Healthy (#cat 636529 #LOT 1405150A) – normal human kidney pooled from a 40-year-old Caucasian female, cause of death: sudden death
Tumor (cat 636632 #LOT 1102289A) – kidney renal cell carcinoma isolated from a 48-year-old male Caucasian.

Supplementary Figure S1

Results of sequencing - PTEN knockout clones

Renca wild-type cell line:

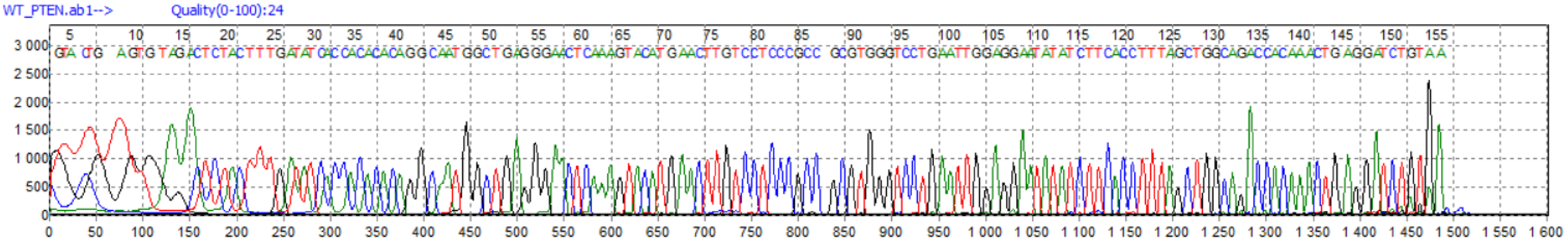

Renca pten/KO (clone 11G4_PTEN):

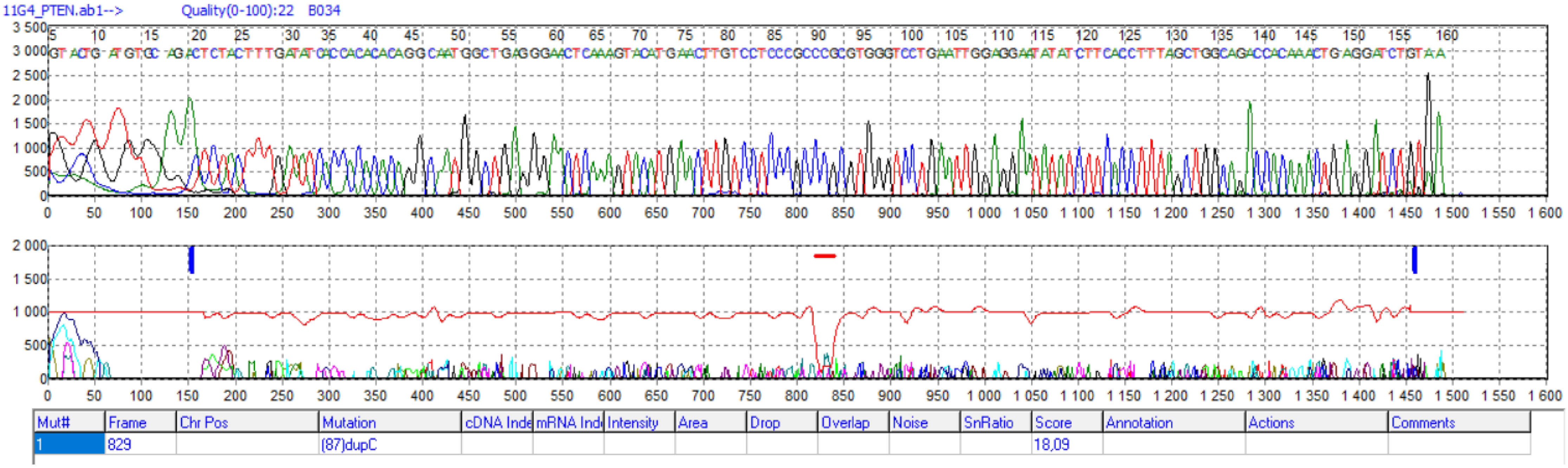

Mut#	Frame	Chr Pos	Mutation	cDNA Inde	mRNA Ind	Intensity	Area	Drop	Overlap	Noise	SnRatio	Score	Annotation	Actions	Comments
1	829		(87)dupC									18,09			

Renca pten/WT – negative control (clone B3B12_PTEN):

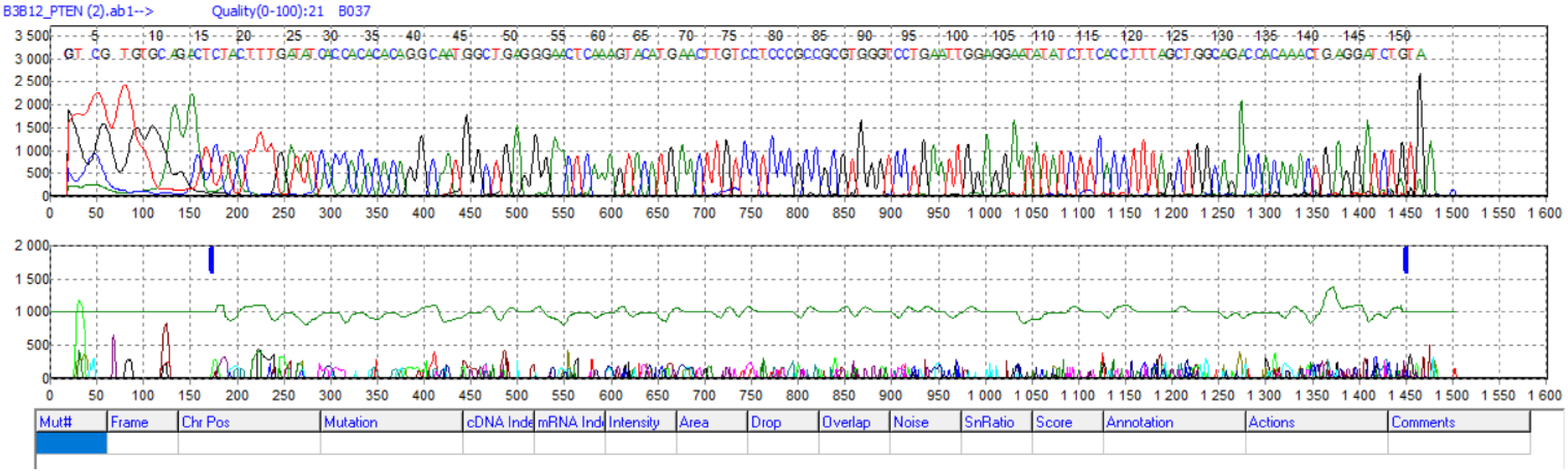

Mut#	Frame	Chr Pos	Mutation	cDNA Inde	mRNA Ind	Intensity	Area	Drop	Overlap	Noise	SnRatio	Score	Annotation	Actions	Comments

☑ Lock Sample File in View

No.	Sample File	Reference File	Dir	Gene	Exon	RF	Start	End	Size	Qualit	Mut#	Mutation1	Mutation2
2	11G4_PTEN.ab1	WT_PTEN.ab1	1-R	Pten	7	2	57840	57974	135	22	1		57905dupG$18
3	B3B12_PTEN (2).ab	WT_PTEN.ab1	1-R	Pten	7	2	57841	57973	133	21	0		
4	sequenc_R11_Synth	WT_PTEN.ab1	1-R	Pten	7	2	57840	57974	135	82	0		
4									466	36	0,50	33,3%	33,3%

Supplementary Figure S2

Size of nanoparticles isolated with the ExoQuick Buffer from Renca cells conditioned medium A) normoxic B) hypoxic, measured using Zeta Viewer Nanoparticle Analyzer C) level of exosomal marker TSG101 in samples of isolated nanoparticles

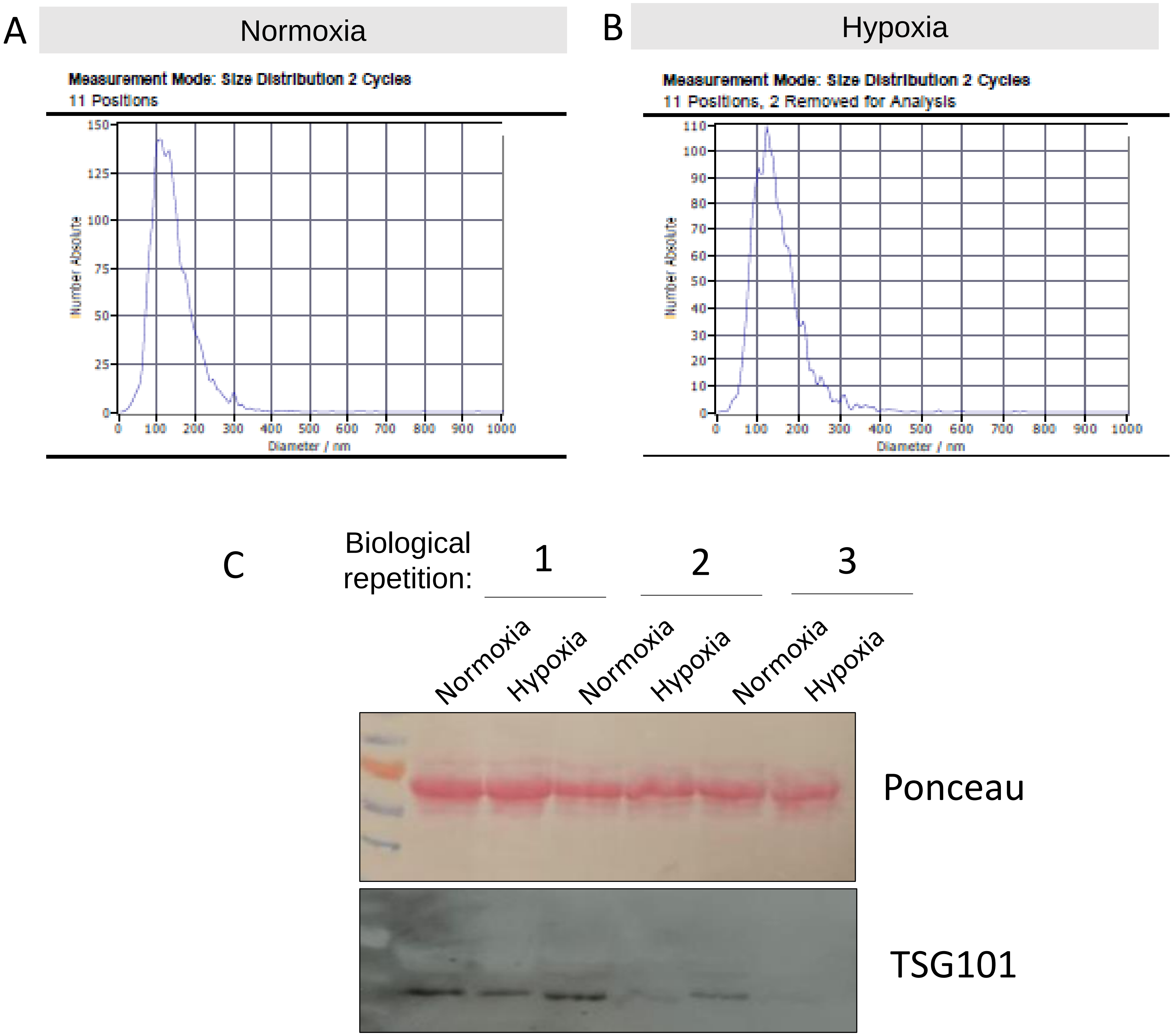

Supplementary Figure S3

Volcano plots of DEmiRNA between Renca pten/WT and pten/KO cell in A) normoxic B) hypoxic conditions. Listed miRNAs significantly changed in pten/KO in vivo samples compared to pten/WT

A Renca pten/WT vs pten/KO normoxic condition

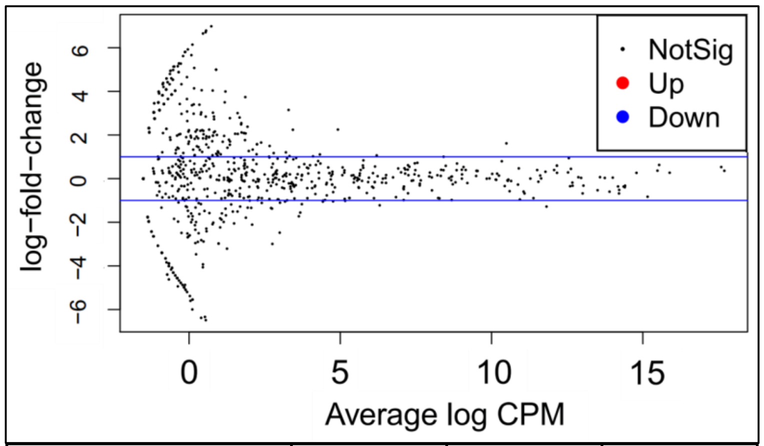

miRNA	logFC	PValue	FDR
**mmu-miR-342-3p**	**4.05**	**0.06**	**1,0000**
mmu-miR-7115-5p	2.97	0.34	1,0000
**mmu-miR-155-5p**	**2.25**	**0.00**	**0,4742**
**mmu-miR-100-5p**	**1.62**	**0.00**	**0,1777**
mmu-miR-3102-3p	1.00	0.06	1,0000
mmu-miR-3068-5p	0.51	0.58	1,0000
mmu-miR-5126	0.00	1.00	1,0000

B Renca pten/WT vs pten/KO hypoxic condition

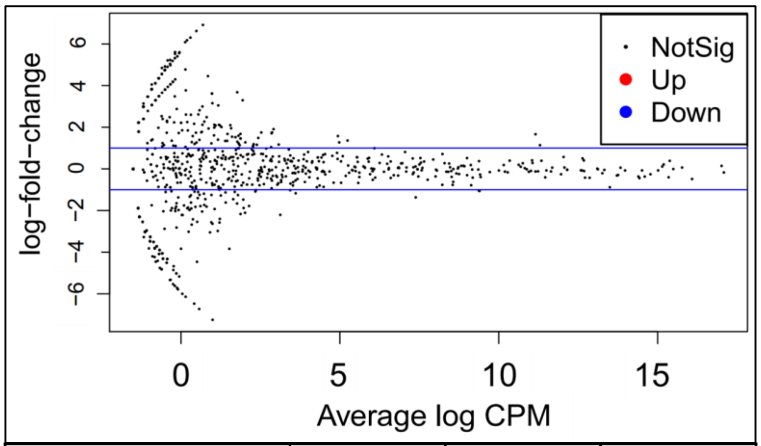

miRNA	logFC	PValue	FDR
**mmu-miR-342-3p**	**6.91**	**0.01**	**1**
**mmu-miR-100-5p**	**1.66**	**0**	**0.1836**
**mmu-miR-155-5p**	**1.58**	**0.01**	**1**
mmu-miR-3068-5p	0.68	0.51	1
mmu-miR-5126	0.04	0.96	1
mmu-miR-7115-5p	-0.67	0.8	1
mmu-miR-3102-3p	-0.77	0.09	1
